# Radiotherapy and precision medicine's role in molecular alterations during chromosomal division: The influence of *MB, TP53, CENPA, BUB1B*, *MAD2L1*, *ZWINT* expression and noncoding RNAs in oral cancer

**DOI:** 10.1016/j.bbrep.2025.102376

**Published:** 2025-11-25

**Authors:** Saloomeh Khoushab, Mina Hobabi Aghmiuni, Anahita Bizhanpour, Behnaz Raei, Saeid Nemati Anaraki, Maliheh Entezari, Afshin Taheriazam, Mehrdad Hashemi

**Affiliations:** a Farhikhtegan Medical Convergence Sciences Research Center, Farhikhtegan Hospital, Faculty of Medicine, TeMS.C., Islamic Azad University, Tehran, Iran; b Department of Genetics, Faculty of Advanced Science and Technology, TeMS.C., Islamic Azad University, Tehran, Iran; c Basic and Molecular Epidemiology of Gastrointestinal Disorders Research Center, Research Institute for Gastroenterology and Liver Diseases, Shahid Beheshti University of Medical Sciences, Tehran, Iran; d Department of Genetic, Faculty of Biological Sciences, NT.C., Islamic Azad University, Tehran, Iran; e Department of Opetative, Faculty of Dentistry, TeMS.C., Islamic AzadUniversity, Tehran, Iran; f Department of Orthopedics, Faculty of Medicine, TMS.C., Islamic Azad University, Tehran, Iran

**Keywords:** Oral cancer, Radiotherapy, Precision medicine, Prognostic markers

## Abstract

Oral cancer, a significant global health concern, remains a challenging disease with high mortality rates. Despite advancements in treatment, late diagnosis and lack of effective biomarkers hinder patient outcomes. To address this, our study investigates the potential of specific genes (*MB, TP53, CENPA, BUB1B, MAD2L* and *ZWINT*) and microRNAs (hsa-mir-607, hsa-mir-556-5p, hsa-mir-1225-3p, hsa-mir-361-3p) as prognostic markers and therapeutic targets for oral cancer. We hypothesize that these genes and miRNAs play crucial roles in regulating cell cycle progression, DNA damage repair, and apoptosis in oral cancer cells. By analyzing their expression patterns in oral cancer tissues and adjacent normal tissues, we aim to identify potential biomarkers for early diagnosis and prognosis. Additionally, we explore the potential of targeting these molecules to enhance the efficacy of radiation therapy and improve patient outcomes. Our study contributes to the comprehension of the molecular processes underlying oral cancer and provides insights into the development of novel therapeutic strategies based on personalized medicine.

## Highlights

• Oral squamous cell carcinoma (OSCC) is a prevalent cancer affecting the mouth.
• Key genes (***MB, TP53****,* ***CENPA****,* ***BUB1B****,* ***MAD2L1****,* ***ZWINT***) and miRNAs (**hsa-mir-607**, **hsa-mir-361-3p**, **hsa-mir-556-5p**, **hsa-mir-1225-3p**) play critical roles in OSCC progression and radiation response.
• Mutations in *MB*, *TP53, CENPA, BUB1B, MAD2L1* and *ZWINT* disrupt genomic stability, leading to poor radiation response.
• miRNAs like hsa-mir-607, hsa-mir-361-3p, hsa-mir-556-5p, and hsa-mir-1225-3p serve as diagnostic biomarkers, influencing tumor radioresistance and therapeutic response.

## Graphical abstract

**Figure:**
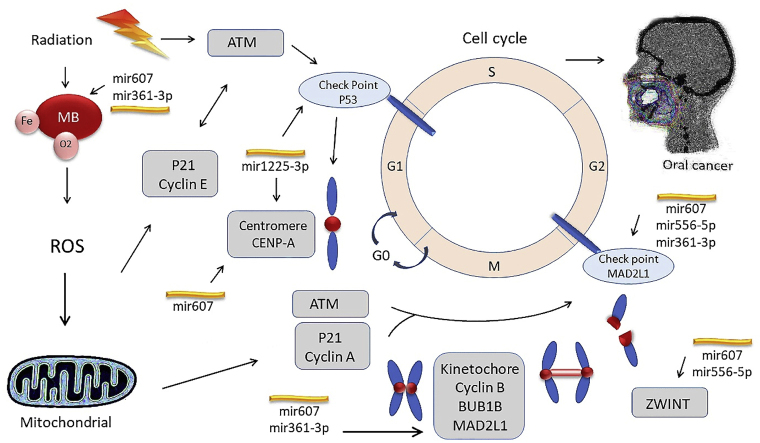

## 1 Introduction

Oral cancer, ranked as the sixteenth most common malignant neoplasm, is a highly prevalent cancer with a significant mortality rate. In 2022, approximately 389,485 new cases and 188,230 deaths from this disease were reported worldwide. Diagnosis is often delayed despite the oral cavity's accessibility for examination. No effective biomarkers for oral cancer diagnosis have been identified to date. The mortality rate remains high despite treatment advancements. Late diagnosis and access to care, particularly in rural areas, significantly contribute to this issue. Consequently, the survival rate is approximately 50 %, with variations across different races and locations. Delayed diagnosis results in poor prognosis and heightened lymph node metastasis [[1], [2], [3], [4], [5]]. To achieve the best results in treating non-metastatic head and neck SCC, a multimodal approach involving surgery and (chemo)radiotherapy or upfront chemo radiotherapy is typically employed, as it surpasses the effectiveness of single-modality therapies [6]. Ionizing radiation is undeniably crucial in managing HNSCC. Many HNSCC patients receive external beam radiation therapy (EBRT). Intensity-modulated radiation therapy (IMRT) is an advanced EBRT technique that precisely targets the tumor or specific areas within it, reducing damage to critical structures and improving the quality of life for patients [7,8]. Interest in brachytherapy as a primary treatment for oral cancer has diminished, largely due to improved surgical techniques, the shift from LDR to HDR (with higher complication risk), and advances in modern EBRT [9]. Proton therapy (PT) provides sharper distal dose fall-off than photons and may support de-escalation strategies (especially in HPV-associated OPC/NPC) and potentially stronger immunogenic effects. DAHANCA-35 (NCT04607694) is a Danish two-parallel-arm randomized program in pharyngeal/laryngeal HNSCC comparing PT vs photons (66–68 Gy in 33–34 fractions) with concurrent chemotherapy. Co-primary endpoints are late grade ≥2 dysphagia and grade ≥4 xerostomia; secondary endpoints cover disease control/survival, toxicity, and patient-reported outcomes [10]. BNCT targets tumor cells loaded with boron; neutron irradiation generates short-range high-LET particles (α, ^7^Li) that kill cancer while sparing nearby tissue. In Takeno et al., 2024 (69 pts), ORR was 80.5 % with 1-year LRC/PFS/OS of 57.1 %/42.2 %/75.4 %; results were better at earlier stages and without prior chemotherapy, supporting BNCT as an option when surgery or conventional RT aren't feasible [11,12]. Carbon-ion radiotherapy (CIRT), with sharp dose fall-off and high linear energy transfer (high RBE), enables dose escalation to tumors while better sparing adjacent normal tissues. Emerging clinical data suggest superior local control with acceptable toxicity—particularly for radioresistant head-and-neck tumors and select cases such as advanced salivary gland and nasopharyngeal cancers—though access remains limited to specialized centers [13].The relationship between single-strand break (SSB) damage and linear energy transfer (LET) is indirect yet critically important, rooted in the complexity of the initial DNA lesion. While all ionizing radiation generates a high yield of SSBs, high-LET radiation (e.g., carbon ions) does not merely produce more of them; it fundamentally alters their nature. The dense ionization tracks characteristic of high-LET particles create clusters of DNA lesions, where multiple SSBs, base damages, and double-strand breaks (DSBs) occur in close proximity. Consequently, what might appear as a simple, isolated SSB in a low-LET context is often a component of a more complex, clustered DNA lesion when induced by high-LET radiation. This clustering has profound implications for repair. The close proximity of lesions can overwhelm the base excision repair (BER) pathway, which is responsible for SSB repair, and can convert otherwise manageable SSBs into more severe DSBs during replication or attempted repair. Therefore, the primary impact of elevated LET is not a quantitative increase in simple SSBs, but a qualitative shift towards complex SSBs and clustered damage sites that challenge canonical repair mechanisms and significantly increase the biological effectiveness of the radiation [14]. When cells are exposed to ionizing radiation, DNA double-strand breaks (DSBs) are induced. ATM is activated, leading to the stabilization of p53, which in turn activates p21. p21 inhibits cyclin-CDK complexes, resulting in cell cycle arrest in the G1 phase. Additionally, ATM can activate p38 MAPK to stabilize p21 mRNA. If DNA damage persists into the S phase, replication fork progression can be disrupted, leading to further DNA damage and chromosomal instability. The G2/M checkpoint prevents cell division until DSBs are repaired. The intricate balance of cell cycle checkpoints ensures proper cell proliferation and prevents the accumulation of genetic damage, which can contribute to cancer development [[15], [16], [17]]. However, as cell carcinogenesis often involves multiple genes interacting within a regulatory network, understanding the full picture requires considering these complex interactions. This perspective is central to modern cancer biology: cancer is not a disease caused by a single faulty gene, but by the collective failure of an entire network. As Venkitaraman (2003) established in his work “A Growing Network of Cancer-Susceptibility Genes,” tumors arise from complex interactions between many genes. This foundational idea directly informs our study. We are not just looking at individual genes like MB, TP53, or CENPA in isolation. Instead, we are investigating how they work together as part of a larger system. The dysregulation of this interconnected network—including the genes we studied and the microRNAs that control them—is what ultimately drives the development of oral cancer. The cancer emerges from the breakdown of the entire team, not just one player [18]. *Myoglobin (MB)* is a protein primarily found in heart and muscle cells. It stores and transports oxygen within these cells. Beyond its oxygen-carrying role, *MB* also helps regulate nitric oxide levels and protect cells from oxidative stress. While traditionally associated with muscle tissue, MB has been increasingly identified in various epithelial tumors and cancer cell lines [[19], [20], [21], [22], [23]]. However, MB expression outside muscle is heterogeneous and context-dependent across tumor types, cohorts, and assay platforms [[23], [24], [25], [26]].In our OSCC cohort, MB was significantly downregulated in tumors versus matched adjacent normal tissues. when cells are exposed to radiation, p53 can induce apoptosis, a programmed cell death process, to eliminate damaged cells. This is particularly important in preventing the accumulation of genetic mutations that can lead to cancer. However, p53 can also promote cell survival under certain conditions, such as low-dose radiation or in cells with DNA repair capabilities. This dual role of p53 in both promoting cell death and survival is crucial for maintaining cellular homeostasis and preventing tumorigenesis [27,28]. *CENP-A* is a distinct variant of histone H3, one of the main components of nucleosomes, which are the fundamental building blocks of chromatin. Unlike other histone types, *CENP-A* is uniquely localized to the centromeres in most eukaryotic cells. Its structural characteristics enable a more flexible binding to DNA within the nucleosome core particle [[29], [30], [31], [32], [33], [34]]. Overexpression *of CENP-A*, both acutely and chronically, in *p53* wild-type cells resulted in a notable increase in the percentage of non-cycling cells, indicating the onset of senescence. This effect was markedly diminished in cells lacking p53. These results imply that *CENP-A* overexpression can induce cell cycle arrest and senescence in a manner that depends on *p53* [35,36]. Incorrect chromosome segregation during cell division, known as aneuploidy, can lead to serious consequences. To prevent this, cells have a built-in checkpoint mechanism called the mitotic spindle assembly checkpoint. This checkpoint relies on a highly conserved group of proteins that operate as a signal transduction system*,* among them; *MAD2L1* plays a critical role in the checkpoint's function*. BUB1* and *BUBR1*, both vital proteins within the mitotic checkpoint, are crucial for accurate cell division. If these proteins are compromised, cells may experience slower growth and increased chromosomal instability. *BUBR1*, in particular, attaches to kinetochores that are either unattached or incorrectly attached, helping to stabilize their interaction with microtubules and ensure proper chromosome alignment during mitosis [37,38]. *ZWINT* performs important roles in maintaining the mitotic cycle and is a recognized member of the kinetochore complex needed for the mitotic spindle checkpoint. By controlling the interaction between ZW10 and centromere complexes during mitosis and mitotic prometaphase, *ZWINT* encodes a protein that is obviously engaged in kinetochore activity [39,40]. MiRNAs are tiny (∼22 nucleotides), non-coding RNA molecules that regulate gene expression. They work by binding to specific messenger RNA (mRNA) molecules, which can lead to their degradation or inhibition of protein production. miRNAs are stable and can be a valuable tool for studying gene expression in various diseases, including cancer. In cancer, miRNAs can be either overexpressed or underexpressed, contributing to tumorigenesis by acting as oncogenes or tumor suppressors [[41], [42], [43], [44]]. MicroRNAs represent a promising therapeutic avenue for enhancing the efficacy of radiotherapy(RT). By analyzing miRNA expression levels in tumor tissues, it's possible to predict a tumor's response to RT, monitor treatment progress, and identify patients at risk of recurrence or metastasis. While research has shown the potential of miRNAs as biomarkers, most studies using patient data are currently focused on assessing tumor aggressiveness and response to RT(44). Personalized radiation therapy, which involves using biomarkers to guide treatment, is a promising approach to improve patient outcomes. By identifying specific molecular targets within tumors, we can enhance the effectiveness of radiation therapy while minimizing side effects. These biomarkers could be used to develop new targeted therapies or to optimize radiation dose and fractionation schedules [45].This study aims to examine the impact of non-coding RNAs hsa-mir607, hsa-miR-556-5p, hsa-mir1225-3p, and hsa-mir-361-3p on the expression levels of these genes.

## 2 Materials and methods

### 2.1 Data analysis

This study aimed to pinpoint the specific genes involved in oral cancer by examining gene expression changes using a vast dataset from The Cancer Genome Atlas (TCGA) (https://ualcan.path.uab.edu/analysis.html). RNA-seq data for OSCC were obtained from the TCGA-HNSC cohort through the Genomic Data Commons (GDC) portal. Only tumors and matched normal samples with complete clinical information were included. Expression data were normalized (RSEM values), and differential expression analysis was performed using the Limma package in R, applying a fold change (FC) ≥ 2 and an adjusted p-value (q-value) ≤ 0.01 based on the Benjamini–Hochberg correction to control for false discovery. To start, miRTarBase was used to find miRNA-target interactions. Strong interactions were supported by reporter assays and validated by methods like Western blot or qPCR.We analyzed the expression levels of these miRNAs in both cancerous and healthy oral tissues. Next, we utilized the TargetScan website to identify potential genes that might be regulated by this miRNA. This step involved searching for genes that are likely to be targeted and influenced by the miRNA's function (https://www.targetscan.org/vert_80/). we focused on the INHBA gene and employed the STRING online database (https://string-db.org/) to retrieve its associated protein-protein interaction (PPI) network. All participating patients provided written informed consent after being informed of the study's purpose. This study was approved by the Islamic Azad University, Tehran Medical Sciences Research Ethics Committee (The number for ethical approval is: IR.IAU.PS.REC.1402.567).

### 2.2 Validation of miRNA expression using miRTarBase

The expression of four miRNAs was validated utilizing miRTarBase, with data collected and analyzed through extensive datasets available on the miRTarBase platform. Specific details about each database are discussed further in the Results section.

### 2.3 Detection and analysis of genetic variants in *MB, TP53, CENPA, BUB1B*, *MAD2L1* and *ZWINT*

RNA-seq data were not generated in this study; instead, publicly available datasets from TCGA and miRWalk were used to obtain transcriptomic and regulatory information for hub gene analysis. mRNAs were identified by intersecting the predicted miRNA target genes and RNA-Seq data from TCGA. Among the identified genes, MB, CENPA, MAD2L1, and ZWINT were selected based on TCGA expression profiles and miRWalk analysis. TP53 and BUB1B were additionally included due to their well-documented biological relevance. Literature evidence indicates that BUB1B interacts with and shows correlated expression patterns with MB, CENPA, MAD2L1, and ZWINT in several cancers, supporting its inclusion as a key hub gene.

### 2.4 Analyzing and predicting the expression of key mitotic checkpoint genes in oral cancer

We utilized the UALCAN (https://ualcan.path.uab.edu/) web resource to extensively analyze HNSC OMICS data focusing on mitotic checkpoint genes. This platform provided comprehensive information on sample sizes, cancer stages, gender distribution, and histological subtypes for *MB, TP53, CENPA, BUB1B*, *MAD2L1*and,*ZWINT* datasets. Our in-depth analysis sheds light on the role and clinical significance of the HNSC gene in Oral Squamous Cell Carcinoma across various clinical and molecular aspects. Furthermore, the Gene Expression Profiling Interactive Analysis (GEPIA, http://gepia.cancer-pku.cn/) platform is an online resource that provides fast and highly customizable analytical functionalities by leveraging data from TCGA and GTEx. GEPIA offers a range of interactive features, including differential expression analysis, profiling visualization, correlation analysis, survival analysis for patients, detection of similar genes, and dimensionality reduction analysis. In this study, GEPIA was utilized to analyze the expression levels of *MB, TP53, CENPA, BUB1B, MAD2L1*, and *ZWINT* genes in head and neck squamous cell carcinoma (HNSC). Our analysis highlights the expression levels of these genes in HNSC.

### 2.5 Tumor purity and gene expression correlation

We looked at immune cell infiltration in relation to mitotic checkpoint genes in Head and Neck Squamous Cell Carcinoma (HNSCC). To achieve this, we used the TIMER database (http://cistrome.org/TIMER/), which includes immune infiltration data for 20 cancer types from The Cancer Genome Atlas (TCGA). We specifically focused on six immune cell types: neutrophils, myeloid dendritic cells, macrophages, CD8^+^ T cells, CD4^+^ T cells, and B cells. Using TIMER's algorithm, we analyzed how the expression of *MB, P53, CENPA, BUB1B*, *MAD2L1* and,*ZWINT* genes correlated with the abundance of these immune cells in HNSCC tumors [46].

### 2.6 Predicting the response to immune checkpoint inhibitor (ICI) treatment in oral cancer

We utilized used two online resources.

• **TISIDB** (http://cis.hku.hk/TISIDB/): This database was used to analyze the relationship between various immune checkpoint inhibitors (ICIs) and HNSCC. ICIs are a promising type of immunotherapy that shows great potential in cancer treatment [47].
• **TIDE algorithm** (http://tide.dfci.harvard.edu/): This algorithm analyzes gene expression data from HNSCC samples to estimate their response to immunotherapy. It considers genes involved in T cell dysfunction and infiltration. A lower TIDE score indicates a more chance of a positive response to immunotherapy with immune checkpoint blockade [48].

### 2.7 Verifying the expression of *MB, TP53, CENPA,BUB1B*,*MAD2L1* and, *ZWINT* genes in oral cancer using RT-qPCR

The validation process for the expression of genes *MB, TP53, CENPA, ZWINT, BUB1B* and *MAD2L1*, along with microRNAs hsa-mir-607, hsa-mir-556-5p, hsa-mir-1225-3p, and hsa-mir-361-3p in HNSCC, followed a series of carefully structured steps. Total RNA was extracted from HNSCC tumor and adjacent margin tissues using the PARSTUS kit (CinnaGen, Iran). Following RNA extraction, cDNA synthesis was conducted using the RevertAid First-Strand cDNA Synthesis Kit (Max Cell, Iran). Subsequently, quantitative RT-PCR was performed in duplicate for each sample using a Real-Time PCR Detection System and SYBR Green Master Mix (Ampliqon, Iran), adhering to the manufacturer's protocol. GAPDH and U6 served as reference genes, and specific primers (detailed in Table 1) were utilized for each target gene.

**Table 1:** The specific primer sequences utilized in this study.

Name	Primer sequence
*MB*- Forward	TTTAGTAGAGGTGGGCAGGAG
*MB*-Reverse	TCGGTTGGGATTTAGAAGAAAGG
*P53*-Forward	ATCTACAAGCAGTCACAGCAC
*P53*-Reverse	TCATAGGGCACCACCACAC
*CENPA*- Forward	ACGCCTATCTCCTCACCTTAC
*CENPA*- Reverse	CAGACAGCATCGCAGAATCC
*ZWINT*- Forward	AGGTGGCAGGCATCTTG
*ZWINT*- Reverse	TGTCGGCTCGTGTCTTC
*MAD2L1*-Forward	TAAATGAATGAGCGTGAGTGTG
*MAD2L1*- Reverse	AGATACTATGGATGGAGGCAAC
*BUB1B*-Forward	TGAGGACCAGCAGACAGC
*BUB1B*-Reverse	AAGAACCAGAGAAGCCAGAGG
GAPDH-Forward	AAGGCTGTGGGCAAGGTCATC
GAPDH-Reverse	GCGTCAAAGGTGGAGGAGTGG
has-mir-607 Forward	AGCGAGGCGTTATAGATCTGG
Steam loop (mir607)	GTCGTATCCAGTGCAGGGTCCGAGGTATTCGCACTGGATACGACGTTCCA
hsa-miR-556-5p Forward	AACACGTGGATGAGCTCATTG
Steam loop (miR-556-5p)	GTCGTATCCAGTGCAGGGTCCGAGGTATTCGCACTGGATACGACCTCATA
Steam loop (mir-1225-3p)	GTCGTATCCAGTGCAGGGTCCGAGGTCCGAGGTATTCGCACTGGATACGACCTGGGGG
Has-mir-1225-3p Forward	AACCATGTGAGCCCCTGTGC
Has-mir-361-3p Forward	AACTTGATTCCCCCAGGTGTGA
Steam loop (mir-361-3p)	GTCGTATCCAGTGCAGGGTCCGAGGTATTCGCACTGGATACGACAAATCAG
U6-Forward	GCTCGCTTCGGCAGCAGCACATATAC
U6-Reverse	CGAATTTGCGTGTCATCCTTGCG
Universal	GTCGTATCCAGTGCAGGGTCC

### 2.8 Patients

**Fig. 1:**
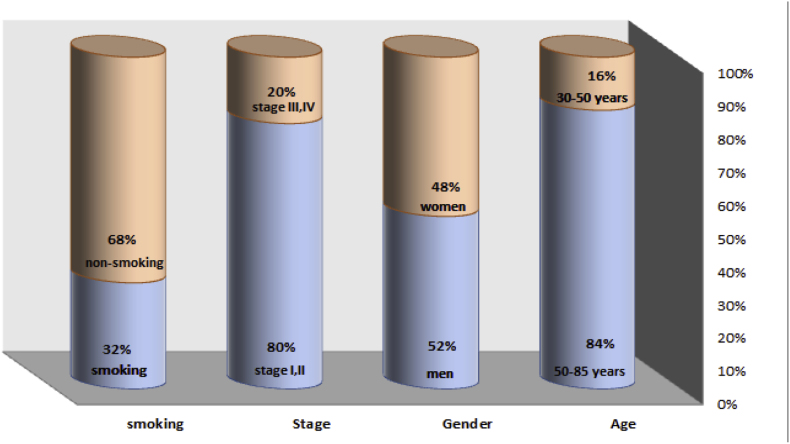
The clinical information, including smoking status, age, stage, and gender, is provided for the samples. In summary, the typical profile in this dataset consists of individuals between the ages of 50 and 85, diagnosed with an early-stage condition, and who are non-smokers.

This study analyzed 50 tissue samples (25 oral cancer, 25 healthy) obtained from patients undergoing surgery at the Imam Khomeini Hospital Cancer Research Center (These patients have different types of oral cancer and all of them generally have oral cancer). Inclusion criteria excluded patients with prior treatment. All procedures were conducted ethically with informed consent and necessary approvals. Fig. 1 provides a summary of the samples' clinical data, while Table 2 provides more specific information.

**Table 2:** This research includes 50 samples, including 25 samples of oral cancer tissue and 25 samples of adjacent normal tissue samples.

Variable		No. (%)
Gender	Male, N (%) Female, N (%)	13 (52 %) 12 (48 %)
Age (years) Stage TNM.T TNM.N	≥50, N (%) <50, N (%) 0, N (%) I, N (%) II, N (%) III, N (%) IVA, N (%) T1, N (%) T2, N (%) T3, N (%) N1, N (%) N2, N (%)	22 (88 %) 3 (12 %) 1 (4 %) 1 (4 %) 4 (16 %) 8 (32 %) 11 (44 %) 1 (4 %) 16 (64 %) 8 (32 %) 7 (28 %) 18 (72 %)

### 2.9 Statistical analysis

We conducted correlation analyses using Spearman's or Pearson's tests. Statistical analyses were performed using R version 4.2.2 and Prism version 10.00. Utilizing R, gene expression analysis (including normalization, filtering, and differential expression analysis) and mutation analysis (including MATH and TMB scores) were performed. Tumor purity, ICI, and TIDE scores were determined using online databases. The 2^−ΔΔCt^ technique was used to quantify the levels of gene expression, and the *t*-test was used to determine significance. The log-rank test was used to assess survival analyses. Prism was used to show the comparisons between groups. Differential gene expression in TCGA samples was analyzed using the linear model method, and significance was determined using FDR adjustment. Other statistical analyses were considered significant with a p-value threshold of <0.05.

## 3 Results

### 3.1 Deregulation of four miRNAs targeted

The miRTarBase platform (miRTarBase) was employed to identify miRNA-target interactions, which were classified as either strong or less strong based on the validation approach. Strong evidence was supported by target site reporter assays and complementary methods like Western blot or qPCR. miRTarBase confirmed strong interactions of hsa-miR-361-3p with SH2B1, Hsa-miR-1225-5p with MT1X, RAP2A, IFI6, FCER1G, and TPN6, Hsa-miR-556-5p with PPP2R2A, and Hsa-miR-607 with FTO (Table 3).

**Table 3:**
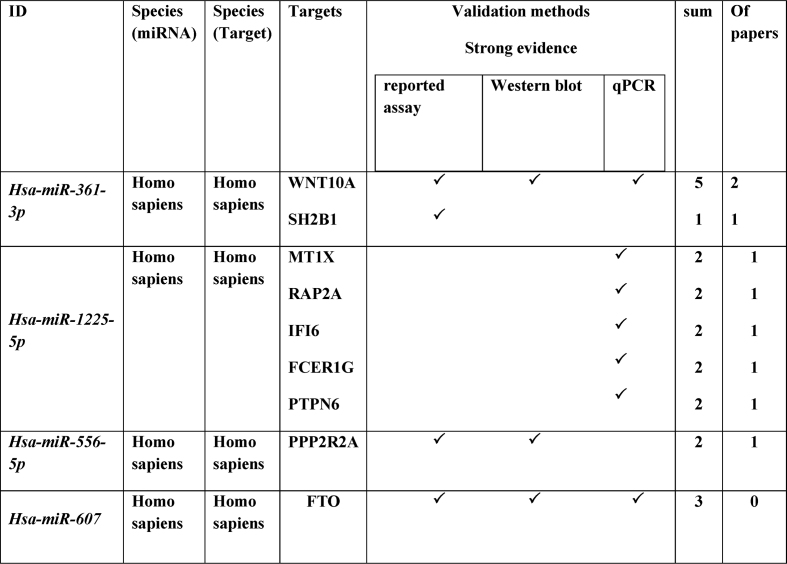
This table presents a list of miRNA-target interactions identified through the miRTarBase platform.

### 3.2 miRNA-mRNA interaction network

From the TCGA and MIRWALK, we extracted 7076 mRNA-miRNA interactions. By identifying 453 mRNAs that were common across these datasets, as depicted in Fig.e 2. Finally, we visualized the resulting network of mRNA-miRNA interactions using the Cytoscape software, as depicted in Fig. 10.

**Fig. 2:**
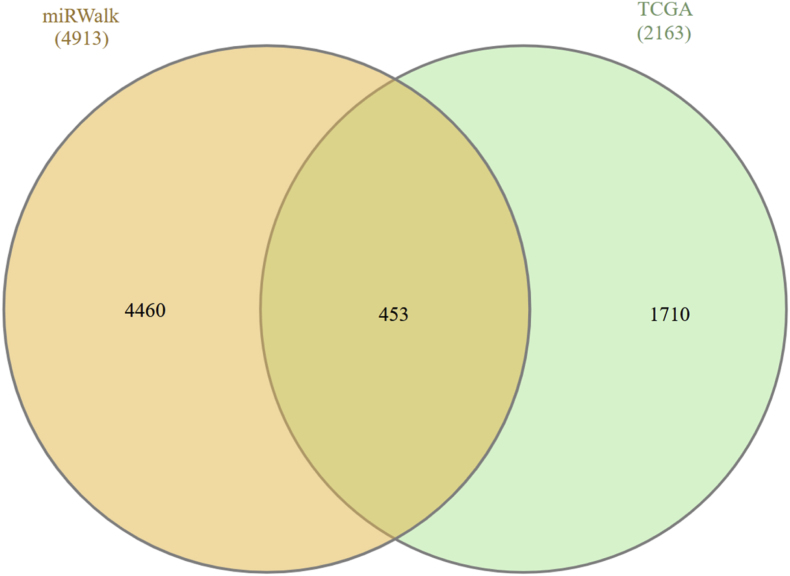
The diagram shows that 453 entries are shared between TCGA (2163 entries) and MIRWALK (4913 entries).

### 3.3 P53 pathway-related gene expression changes in oral cancer

**Fig. 3:**
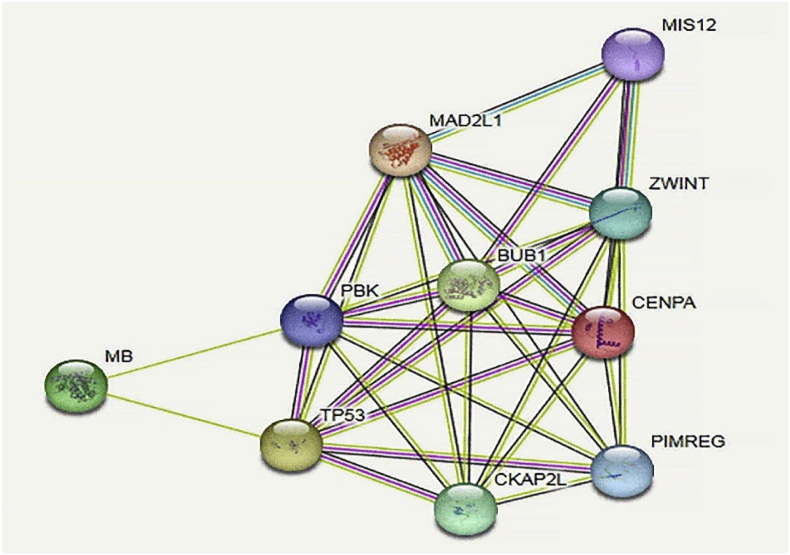
STRING-based PPI network of the MB, P53, CENPA, BUB1B, MAD2L1, and ZWINT genes. This diagram shows the gene network, with BUB1 standing out as a major hub, having the most connections to other genes. Crucially, BUB1 plays a vital role in cell division by helping to form the mitotic spindle, the structure that ensures chromosomes are correctly separated during cell division. This makes it essential for preventing errors in the process.

We began our analysis by retrieving a head and HNSC dataset from TCGA database and performing normalization procedures to ensure data consistency. Next, we leveraged the Reactome database to identify all genes associated with the p53 pathway. With this set of genes in hand, we conducted a comprehensive enrichment analysis using STRINGdb, exploring the functional relationships between these genes. The key findings from this analysis are presented visually in Fig. 3.

### 3.4 Genetic mutations in *MB, TP53, CENPA,BUB1B*,*MAD2L1* and *ZWINT* genes in high and low expression groups

**Fig. 4:**
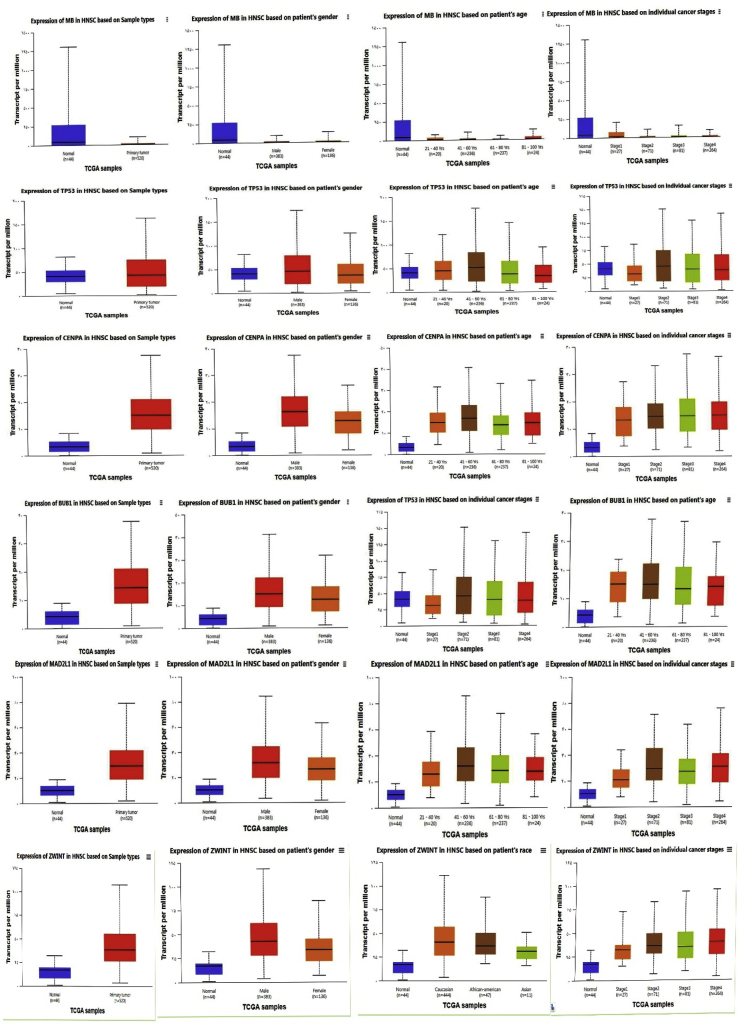
The expression levels of the MB, TP53, CENPA, BUB1B, MAD2L1, and ZWINT genes were analyzed using data from TCGA-HNSC. **MB gene**: Shows a decrease in activity, which becomes even more pronounced in higher-grade, more advanced tumors. **Other key genes** (MAD2L1, CENPA, TP53, ZWINT, BUB1): These genes show increased activity in tumors. Similar to the MB gene, this effect is amplified in higher-grade tumors, meaning their activity levels rise even further as the cancer advances. In simple terms, as the tumor becomes more severe, the activity levels of these genes become more extreme—those that are high increase even more, and those that are low decrease further.

**Fig. 5:**
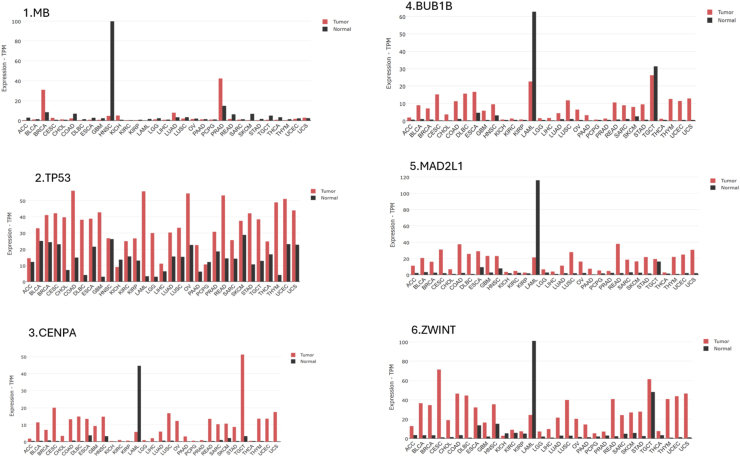
The expression levels of the MB, TP53, CENPA, BUB1B, MAD2L1, and ZWINT genes were analyzed using data from GEPIA. This figure displays distinct gene expression patterns within a cancer consensus panel: The MB gene shows consistently reduced expression across most cancer samples. In contrast, the other panel genes—MAD2L1, CENPA, TP53, ZWINT, and BUB1—demonstrate increased expression.

**Fig. 6:**
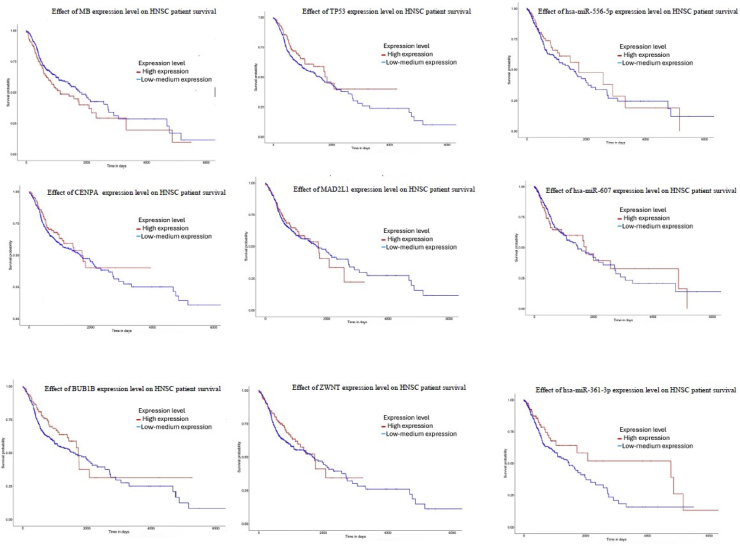
The figure illustrates how the expression levels of MB, TP53, CENPA, BUB1B, MAD2L1, ZWINT, hsa-miR-607, hsa-miR-361-3p, and hsa-miR-556-5p are associated with the survival outcomes of patients with HNSC.

**Fig. 7:**
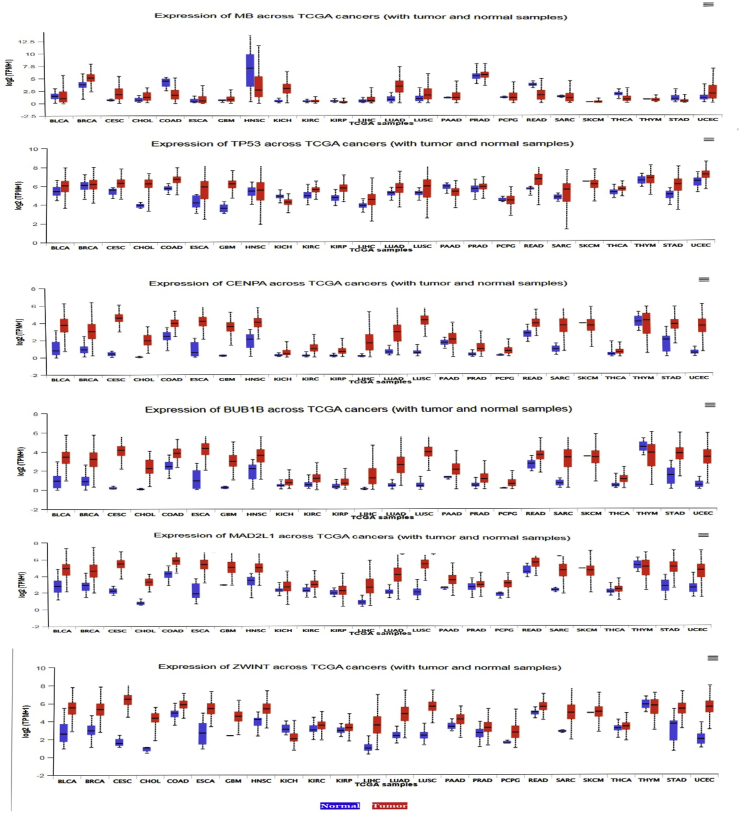
Comprehensive cancer analysis using the TCGA database for MB, CENPA, TP53, BUB1B, MAD2L1, and ZWINT. The MB gene is consistently under-expressed in tumors, while other panel genes (MAD2L1, CENPA, TP53, ZWINT, BUB1) are over-expressed.

Based on TCGA and miRWalk analyses, MB, CENPA, MAD2L1, and ZWINT were identified as potential key genes. In addition, TP53 and BUB1B were included as hub genes due to their established biological relevance and previously reported associations with the aforementioned genes in various cancers [5,[49], [50], [51]]. We employed the UALCAN and GEPIA database to evaluate the expression patterns of *MB, TP53, CENPA, BUB1B*, *MAD2L1,* and *ZWINT* genes across various clinical factors in HNSC. The simultaneous analysis across two databases and the consistency of the results enhance the reliability and credibility of the study. Fig. 4, Fig. 5 depict these results, especially in these 2 UALCAN and GEPIA databases, revealing a consistent decrease in *MB* expression and an increase in *TP53, CENPA, BUB1B*, *MAD2L1*and *ZWINT* expression. Furthermore, These findings suggest that these genes, particularly *MB, TP53, CENPA, BUB1B*, *MAD2L1*and *ZWINT* may serve as potential diagnostic biomarkers for HNSC, as their expression levels are elevated across different stages, subtypes, and genders. The UALCAN online database was used to investigate the association between the expression levels of hsa-mir-607, hsa-mir-361-3p, and hsa-mir-556-5p and the survival of patients with HNSC. The results of this analysis are shown in Fig. 6. No data were available for hsa-mir-1225-3p. Fig. 7 presents a pan-cancer analysis from the TCGA database, highlighting the genomic and cellular variations and commonalities across different tumor types for *MB, TP53, CENPA, BUB1B, MAD2L* and *ZWINT*.

### 3.5 The relationship between the expression levels of *MB, TP53, CENPA, BUB1B* and *MAD2L1* and the purity of tumors and ICI

**Fig. 8:**
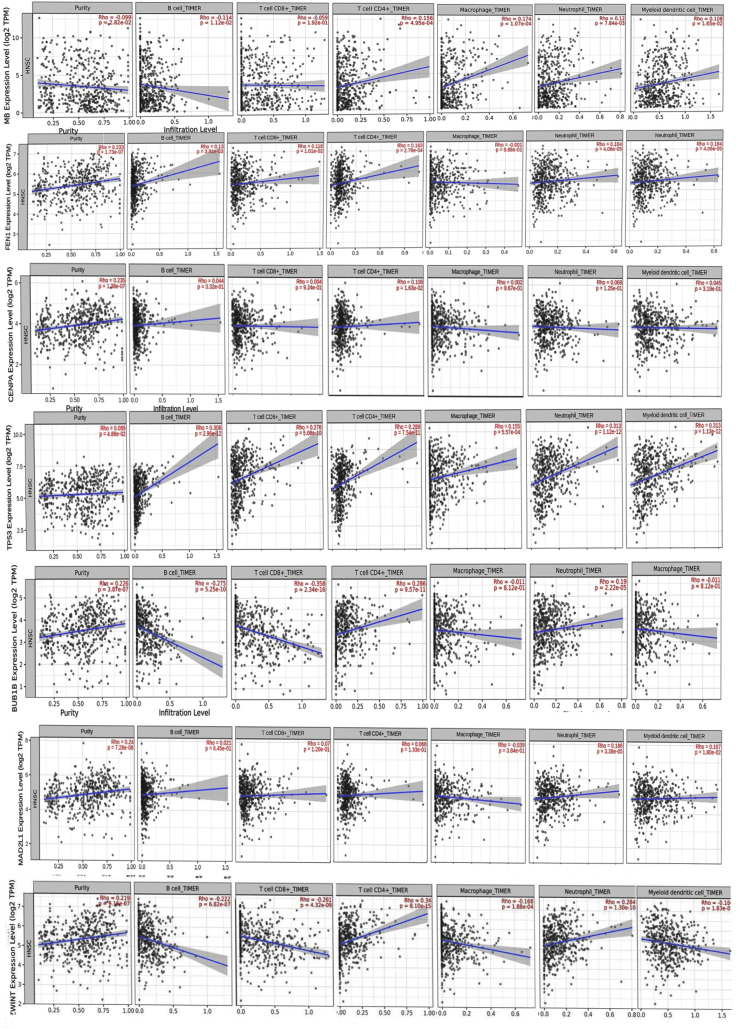
Correlation between immune marker sets and tumor markers (MB, TP53, CENPA, BUB1B, MAD2L1, ZWINT) in HNSC. This figure reveals significant correlations between the studied gene panel and key immune cell markers in the tumor microenvironment. The genes exhibit distinct expression patterns linked to various immune cell populations, including innate immune cells (neutrophils, dendritic cells, macrophages) and adaptive immune cells (B-cells, CD8^+^ T-cells, CD4^+^ T-cells).

**Fig. 9:**
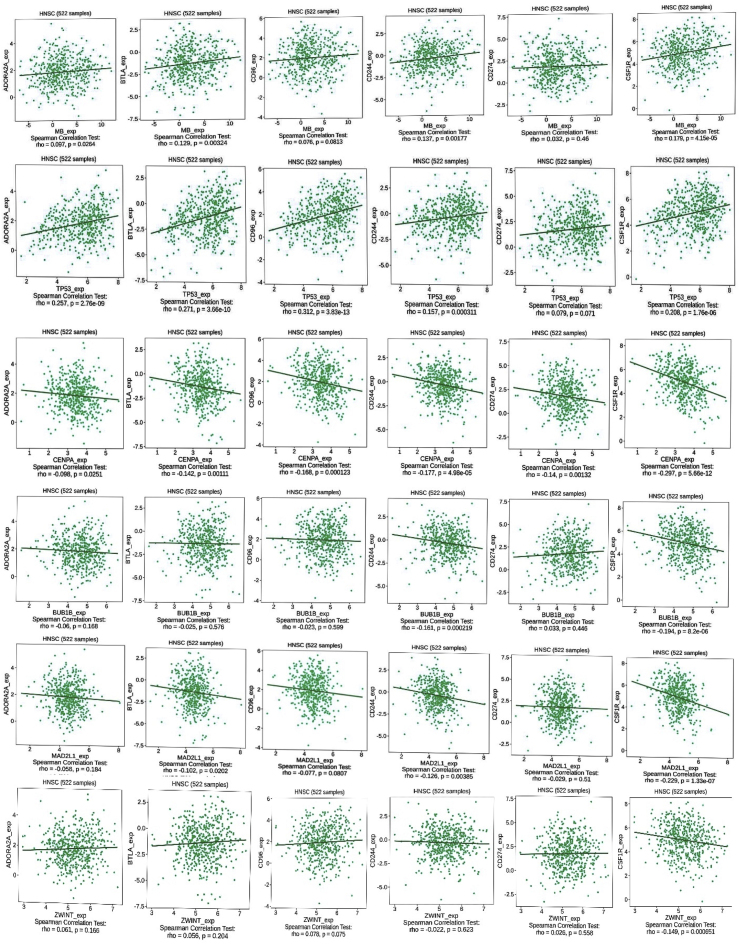
Correlation analysis of MB, TP53, CENPA, BUB1B, MAD2L1, and ZWINT with immune checkpoint inhibitors (ICIs) in TISIDB.

By applying the TIMER method, we examined the association between tumor purity and the expression of *MB, TP53, CENPA, BUB1B*,*ZWINT* and *MAD2L1* genes in HNSC (MB: Rho = −0.099, p = 2.82e-02; *CENPA*: Rho = 0.235, P = 1.38e-07; *TP53*: Rho = 0.089, P = 4.88e-02; *BUB1B***:** Rho = 0.226, P = 3.87e-07; *MAD2L1*: Rho = 0.24; P = 7.28e-08; *ZWINT*: Rho = 0.219,P = 9.16e-07). These findings further support our observations, indicating that the expression levels of *MB, TP53, CENPA, BUB1B, MAD2L1* and *ZWINT* may modulate the tumor microenvironment of HNSC tumors, potentially leading to alterations in the infiltration of various immune cell types. These results are visualized in Fig. 8. Employing the TISIDB database, we investigated the association between the expression levels of *MB, TP53, CENPA, BUB1B*, *MAD2L1*, *ZWINT* and six ICIs in CRC. Our findings indicated a positive correlation between *MB* and *TP53* with ADORA2A, BTLA, CD96, CD244, CD274, and CSF1R. In contrast, *TP53* and *MAD2L1* exhibited a negative correlation with these ICIs. *BUB1B* demonstrated no significant correlation with these ICIs. Fig. 9 illustrates these results.

**Fig. 10:**
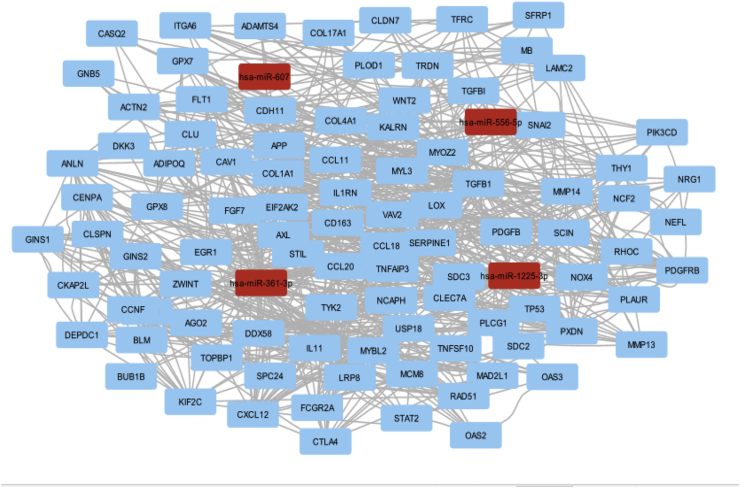
The bipartite gene-mRNA network is illustrated, where MB, TP53, CENPA, BUB1B, MAD2L1, and ZWINT are highlighted in pink, hsa-miR-607, hsa-miR-361-3p, hsa-miR-556-5p, and hsa-miR-1225-3p are shown in blue, and all other elements are represented in gray.

### 3.6 miRNA-mRNA bipartite network

We obtained all interactions between MIRNAs and GENEs from the Ncpath database, identifying 7076 interactions related to the proposed miRNAs. Among these, we found that 453 mRNAs were common to *MB, TP53, CENPA, BUB1B, MAD2L, ZWINT,* hsa-mir-607, hsa-mir-361-3p, hsa-mir-556-5p, and hsa-mir-1225-3p in oral cancer tissues. Subsequently, we illustrated the GENEN-mRNA bipartite network, as shown in Fig. 10.

### 3.7 Examining the expression patterns of *MB, TP53, CENPA, BUB1B, MAD2L1*, *ZWINT* and miRNA network interactions in oral tissues

**Fig. 11:**
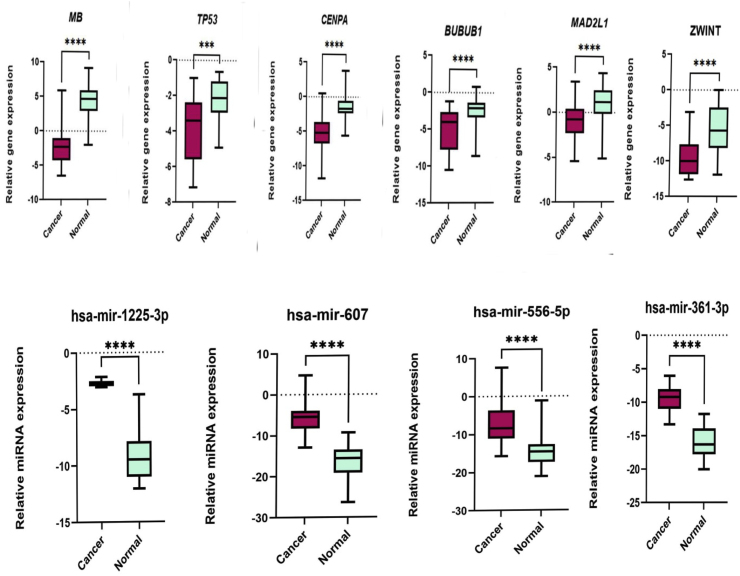
The figure presents the results of the *t*-test analysis conducted on the RT-qPCR data. It shows opposite expression patterns in oral cancer tissues compared to normal tissues. Gene expression: Key genes (MB, TP53, CENPA, BUB1, MAD2L1, ZWINT) exhibit decreased activity in tumors. MicroRNA expression: In contrast, specific microRNAs (hsa-miR-607, hsa-miR-361-3p, hsa-miR-556-5p, hsa-miR-1225-3p) exhibit increased activity in tumors.

**Fig. 12:**
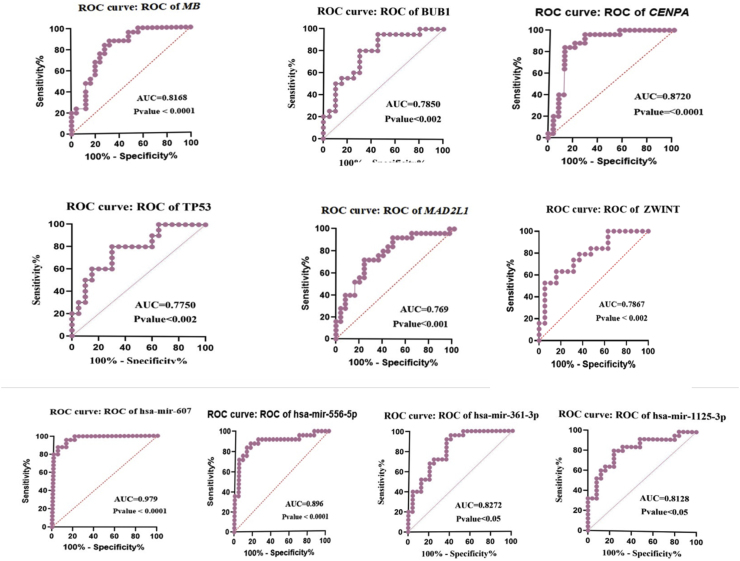
The image presents the receiver operating characteristic (ROC) curve analysis, highlighting the AUC along with the differential expression levels of MB, TP53, CENPA, BUB1B, MAD2L, ZWINT, hsa-miR-607, hsa-miR-361-3p, hsa-miR-556-5p, and hsa-miR-1225-3p.

Given the increasing recognition of *MB, TP53, CENPA, BUB1B, MAD2L* and *ZWINT* as promising biomarkers for predicting patient outcomes, modulating immune responses, and guiding treatment strategies in oral cancer, we conducted a thorough investigation of their expression patterns in 25 oral cancer tissues and 25 adjacent normal tissues using RT-qPCR. Our results, illustrated in Fig. 11., revealed a substantial upregulation of *MB, TP53, CENPA, BUB1B, MAD2L*, *ZWINT*, hsa-mir-607, hsa-mir-361-3p, hsa-mir-556-5p, and hsa-mir-1225-3p in oral cancer tissues compared to normal tissues. These findings were statistically significant, with correlation coefficients exceeding the threshold of P < 0.05, as determined by *t*-test analysis using GraphPad Prism. Fig. 12 displays the receiver operating characteristic (ROC) curve analysis, including the area under the curve (AUC), based on RT-qPCR data. This analysis highlights the differential expression levels of *MB, TP53, CENPA, BUB1B, MAD2L*, *ZWINT*, hsa-mir-607, hsa-mir-361-3p, hsa-mir-556-5p, and hsa-mir-1225-3p between cancer and normal samples. The expression changes observed in all markers in this study were not significantly associated with age and gender.

## 4 Discussion

Oral squamous cell carcinoma (OSCC), the most common type of oral cancer, ranks as the sixteenth most frequent cancer globally, with over 200,000 new cases diagnosed annually. OSCC is divided into three subtypes: buccal mucosal SCC (BMSCC), tongue SCC (TSCC), and lip SCC (LSCC) [[52], [53], [54], [55]]. Radiation exposure in cells can cause DNA damage through reactive oxygen species (ROS), causing lethal double-strand breaks (DSBs). The number of DSBs increases with radiation dose, causing DNA damage that can be directly or indirectly caused by charged particles [16,56]. Cell cycle progression involves G0, G1, S, G2, and M phases, regulated by CDKs and cyclins. Checkpoints monitor DNA synthesis and segregation, with ATM protein activating checkpoints in radiation exposure [15]. MiRNAs are promising diagnostic biomarkers for cancer diagnosis and prognosis, with potential therapeutic applications for enhancing cancer treatments by restoring normal expression patterns or modifying levels [57,58]. MiRNAs can improve radiation therapy effectiveness by profiling tumor radioresistance, monitoring response, and predicting final response and recurrence risk. Despite challenges like conflicting findings and a focus on patient data, their clinical potential remains significant [59]. Myoglobin, a protein primarily found in muscle tissue, was traditionally thought to serve as an oxygen storage molecule. However, recent studies have shown that myoglobin can be expressed in non-muscle tissues, including cancer cells. Interestingly, in head and neck squamous cell carcinoma, higher levels of myoglobin expression have been linked to a more favorable prognosis [19,24,25,60]. Hypoxia, a low oxygen level, hinders cancer radiotherapy effectiveness due to its role in DNA damage. Strategies to increase oxygen levels include using oxygen carriers like perfluorocarbons and hemoglobin-based nanoparticles. These nanoparticles deliver oxygen directly to hypoxic tumor regions, enhancing radiation and chemotherapy treatments' effectiveness [[61], [62], [63]]. Myoglobin expression leads to mitochondrial hyperfusion, a process where mitochondria fuse together. This hyperfusion is associated with cell cycle arrest and inhibition of cell proliferation [64]. *CENP-A* is a crucial protein for proper cell division. While it's primarily known for its role in centromere function during mitosis, recent research suggests it also plays a role in cancer development. Abnormal *CENP-A* expression can lead to changes in chromatin organization, affecting both genome stability and gene expression. These alterations can contribute to cancer progression and the development of metastatic properties [65]. *CENP-A* overexpression makes cells more sensitive to radiation, but this effect is not caused by changes in the DNA and can be reversed. Further analysis using single-cell RNA sequencing showed that long-term CENP-A overexpression can cause many cells with normal *p53* to permanently stop dividing. Our results suggest that *CENP-A* overexpression can disrupt cell division and increase chromosomal instability [66]. Previous studies on OSCC patients treated with a combination of chemotherapy, surgery, and radiation therapy have shown that high levels of *p53* overexpression (over 50 %) often indicate the presence of genetic mutations. Tumors with this *p53* pattern tend to respond poorly to treatment and have a worse prognosis [67]. Multiple kinases, including *BUB1*, play a role in both DNA repair and cell cycle checkpoints. *BUB1* is a key kinase involved in regulating the G2/M cell cycle checkpoint and ensuring accurate chromosome segregation during cell division. Disruptions or mutations in *BUB1* can lead to chromosomal instability and contribute to the development of various cancers [[68], [69], [70]]. *BUB1B* highlights the potential of these hub genes as novel biomarkers. The regulatory network of miRNAs and transcription factors (TFs) demonstrates a strong association between miRNAs and *BUB1B* genes [71]. *MAD2L1* is a protein that plays a crucial role in cell division. It is part of a complex of proteins that ensures accurate chromosome segregation during cell division. The gene that provides the instructions for making *MAD2L1* is located on chromosome 4 in humans [37]. In this study, we observed that radiotherapy-induced up-regulation of hsa-mir-607 and hsa-mir-361-3p was associated with reduced *MB* expression, changes in oxygen levels, and a subsequent decrease in *CENPA* expression, which ultimately disrupted the cell division process. Additionally, hsa-mir-607 and hsa-mir-1225-3p demonstrated increased expression in response to radiotherapy, further contributing to the reduction in *CENPA* expression. Furthermore, decreased expression of *CENPA* directly and *MB* indirectly led to a reduction in *P53* expression. Additionally, radiotherapy directly impacted *TP53* expression by influencing mir-1225-3p.

Mutations in *TP53* are linked to tumor suppressor gene mutations, indicating a poor response to sequential combination therapies. The rate of cellular responses to stressors such as DNA damage, nutrient deprivation, and low oxygen levels (hypoxia) is crucial, as it activates genes that regulate the cell cycle and apoptosis. This function is closely related to the sensitivity of tumors to radiotherapy and chemotherapy [72,73]. Our findings indicate that *TP53* mutations impact *BUB1B*, resulting in reduced expression levels. Moreover, radiotherapy influences the expression of mir-607 and mir-361-3p, causing an increase in their levels and subsequently affecting *BUB1B*. Lower *BUB1B* expression can impair chromosome attachment to the mitotic spindle, leading to errors during cell division. Increased expression of miR-556-5p, miR-607, and miR-361-3p led to decreased expression of *MAD2L1*, a gene essential for the spindle assembly checkpoint. This disruption prevents the cell from arresting in metaphase until all chromosomes are correctly aligned. A malfunctioning kinetochore is one potential cause of chromosome instability (CIN), which can result in aneuploidy. The kinetochore, a complex structure composed of proteins and centromeric DNA, plays a crucial role in segregating sister chromatids into daughter cells by interacting with the mitotic spindle ionizing radiation can induce CIN by causing DNA damage, particularly double-strand breaks. This damage can lead to chromosomal abnormalities and mitotic errors, ultimately contributing to genomic instability and aneuploid [74,75]. Our findings suggest that the role of p53 in oral cancer is not static, but rather a network dynamic: on the one hand, it is repressed by miRNAs, which may contribute to a “loss of function” state. On the other hand, it can be activated by potent stimuli such as CENPA and radiotherapy to activate antitumor responses. These two roles are not contradictory, but rather two different operational states for this key protein, driven by opposing signals in the tumor microenvironment. Consequently, the survival of the cancer cell versus its death depends on the balance between these inhibitory and activating signals. This analysis highlights the remarkable complexity of p53 and the importance of considering it in a systems biology network, rather than a single-agent view.Recent studies indicate that FLASH-seq (FS) is an advanced full-length single-cell RNA sequencing (scRNA-seq) method offering higher sensitivity and faster processing than Smart-seq3. The entire FS protocol can be completed in approximately 4.5 h, is easily automatable and miniaturizable, and employs unique molecular identifiers (UMIs) for accurate molecule counting while reducing strand-invasion artifacts. Consequently, FS provides an efficient approach for high-resolution gene expression profiling across multiple samples [76]. FLASH radiotherapy, which delivers radiation at ultra-high dose rates (>40 Gy s^−1^), selectively destroys tumor cells while sparing normal tissue. At such dose rates, the formation yield of hydroxyl-radical products decreases as the dose rate increases, indicating reduced reactive-oxygen-species production. This effect is attributed to transient oxygen depletion, which limits oxidative damage in healthy tissues while maintaining lethal effects in tumors [77]. These findings collectively highlight that both technological and radiobiological advancements—such as the enhanced resolution of FLASH-seq for transcriptomic profiling and the oxygen-depletion effect observed in FLASH radiotherapy—contribute to a deeper understanding of radiation responses at the molecular level. Together, they underscore the potential of integrating high-throughput single-cell sequencing with precision radiotherapy to elucidate gene-expression dynamics and optimize therapeutic strategies for cancer treatment. *ZWINT*, a gene responsible for sustaining the mitotic cycle and a known component of the kinetochore complex required for the mitotic spindle checkpoint, was expressed less when miR-556-5p and miR-607 were expressed more. We identified hsa-mir-607, hsa-mir-361-3p, hsa-mir-556-5p, and hsa-mir-1225-3p as independent diagnostic biomarkers for OSCC. In conclusion, it appears that *MB, TP53, CENPA, BUB1B, MAD2L*, *ZWINT*, hsa-mir-607, hsa-mir-361-3p, hsa-mir-556-5p, and hsa-mir-1225-3p plays a crucial role as mediators in cancer progression, influencing OSCC development through complex mechanisms. We identified hsa-mir-607, hsa-mir-361-3p, hsa-mir-556-5p, and hsa-mir-1225-3p as independent diagnostic biomarkers for OSCC. In conclusion, it appears that *MB, TP53, CENPA, BUB1B*, *MAD2L, ZWINT*, hsa-mir-607, hsa-mir-361-3p, hsa-mir-556-5p, and hsa-mir-1225-3p play a crucial role as mediators in cancer progression, influencing OSCC development through complex mechanisms. MicroRNAs offer a promising avenue for enhancing the effectiveness of radiotherapy. By analyzing miRNA expression patterns, we can potentially predict how tumors will respond to radiation therapy before treatment begins. Furthermore, monitoring miRNA levels during treatment can provide valuable insights into treatment response and guide treatment adjustments. This information can also help predict the likelihood of tumor recurrence or metastasis. MicroRNAs hold significant clinical potential for improving radiotherapy outcomes. They can serve as valuable biomarkers to predict tumor radioresistance, monitor treatment response, and assess the risk of recurrence or metastasis. While promising, current research primarily focuses on predicting tumor aggressiveness, with limited focus on individual patient responses.Several studies have demonstrated that specific miRNAs, such as hsa-mir-607, hsa-mir-556-5p, hsa-mir1225-3p, hsa-mir-361-3p, are dysregulated in oral squamous cell carcinoma and correlate with radiosensitivity or radioresistance. Monitoring these miRNAs during radiotherapy can provide insights into individual tumor responses and guide treatment adjustments, highlighting their potential as biomarkers in oral cancer management [[78], [79], [80]].

In the end Our study demonstrates that the dysregulation of p53 and its interactive network with key microRNAs serves as a critical determinant of radiosensitivity in oral cancer. The identified expression signatures correlate significantly with patient response to radiotherapy, which functions primarily through oxidative stress pathways. This suggests that pre-therapeutic profiling of this p53-centric network could provide a valuable predictive biomarker.

By analyzing these molecular alterations prior to biopsy, clinicians may better stratify patients, potentially identifying those who would benefit from treatment intensification or novel sensitizing agents. While further validation in larger cohorts is warranted, our findings highlight the promise of targeting the p53-miRNA axis to overcome radioresistance and personalize therapeutic strategies in oral oncology.

## 5 Conclusion

This review highlights the intricate interplay of various cellular and molecular mechanisms in the development and progression of OSCC. The findings underscore the critical roles of key genes and proteins, including *MB, TP53, CENPA, BUB1B, MAD2L* and *ZWINT* in regulating cell cycle progression, DNA damage repair, apoptosis, cell cycle Arrest and tumor suppression. These findings underscore the importance of understanding the complex regulatory networks involved in OSCC development and the potential of targeting these pathways for therapeutic intervention. Furthermore, the study emphasizes the potential of miRNAs as valuable biomarkers for predicting tumor response to radiotherapy, monitoring treatment progress, and assessing the risk of recurrence. While significant progress has been made, further research is crucial to translate these findings into clinically relevant applications for improving the management and treatment of OSCC patients.

## Ethics approval and consent to participate

All participating patients provided written informed consent after being informed of the study's purpose. This study was approved by the Islamic Azad University, Tehran Medical Sciences Research Ethics Committee (The number for ethical approval is: IR.IAU.PS.REC.1402.567).

## Funding

None.

## CRediT authorship contribution statement

**Saloomeh Khoushab:** Investigation. **Mina Hobabi Aghmiuni:** Investigation. **Anahita Bizhanpour:** Investigation. **Behnaz Raei:** Software, Validation. **Saeid Nemati Anaraki:** Project administration. **Maliheh Entezari:** Project administration, Writing – original draft. **Afshin Taheriazam:** Data curation, Writing – original draft. **Mehrdad Hashemi:** Conceptualization, Supervision, Writing – original draft.

## Declaration of competing interest

The authors declare that they have no known competing financial interests or personal relationships that could have appeared to influence the work reported in this paper.

## Data Availability

No data was used for the research described in the article.
